# Supplementary material for: Holocene seasonal temperature evolution and spatial variability over the Northern Hemisphere landmass
Source: Nat Commun. 2022 Sep 10;13:5334. doi: 10.1038/s41467-022-33107-0 (PMC9464234; doi:10.1038/s41467-022-33107-0)
Supplement: Supplementary file 1 — Supplementary Information [file 41467_2022_33107_MOESM1_ESM.pdf]

Supplementary Information for

Holocene seasonal temperature evolution and spatial variability over the Northern Hemisphere landmass

by Wenchao Zhang *et al*.

This PDF file includes:

Supplementary Figures 1 to 9

Supplementary Tables 1 to 2

Supplementary Data 1 to 3

Supplementary References

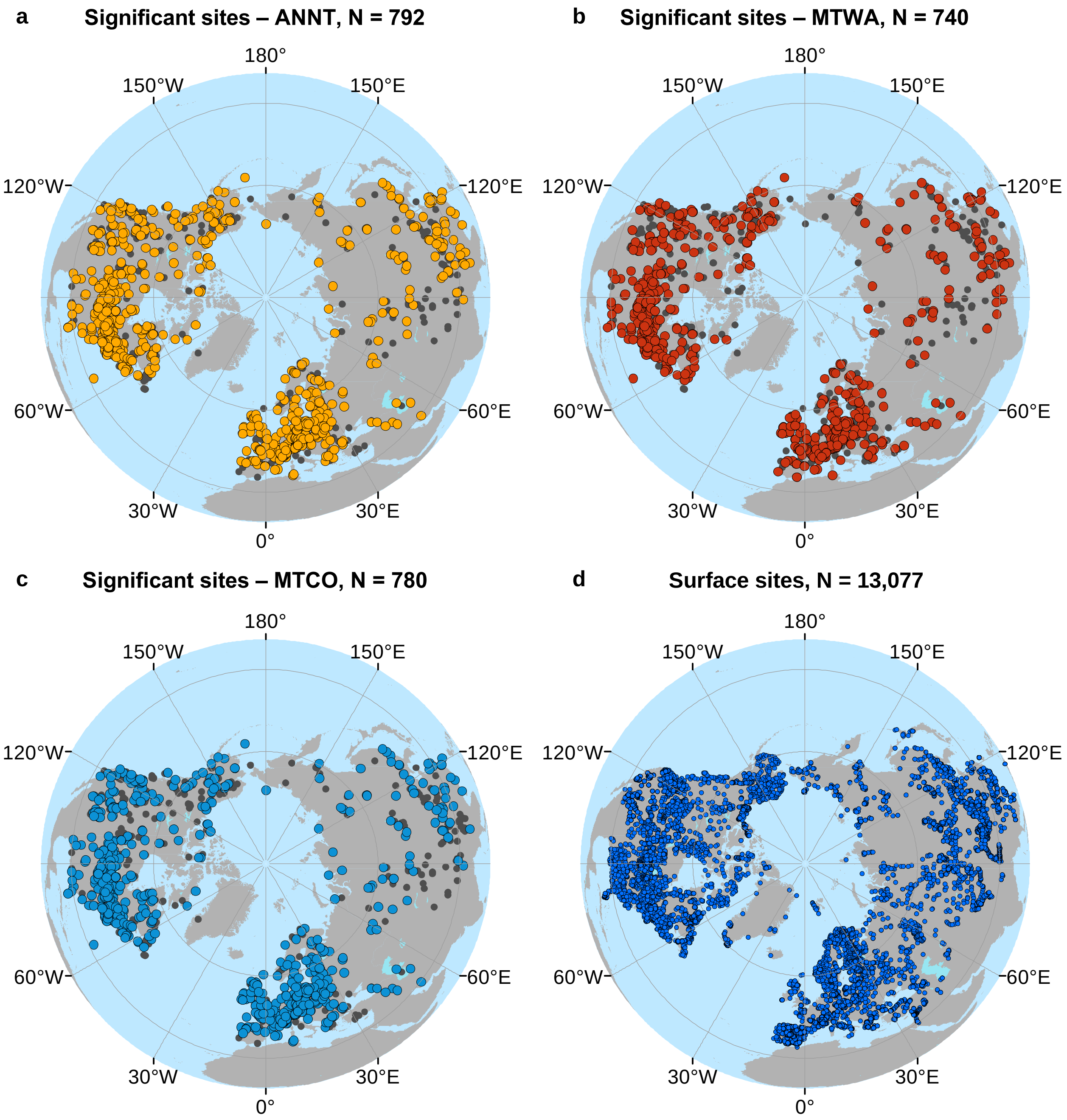

Supplementary Fig. 1. Holocene and modern pollen site distributions. a–c, Maps showing the significant (coloured circles) and all selected (smaller grey circles) records of ANNT (**a**), MTWA (**b**) and MTCO (**c**). **d**, Surface pollen sites for pollen–climate calibration.

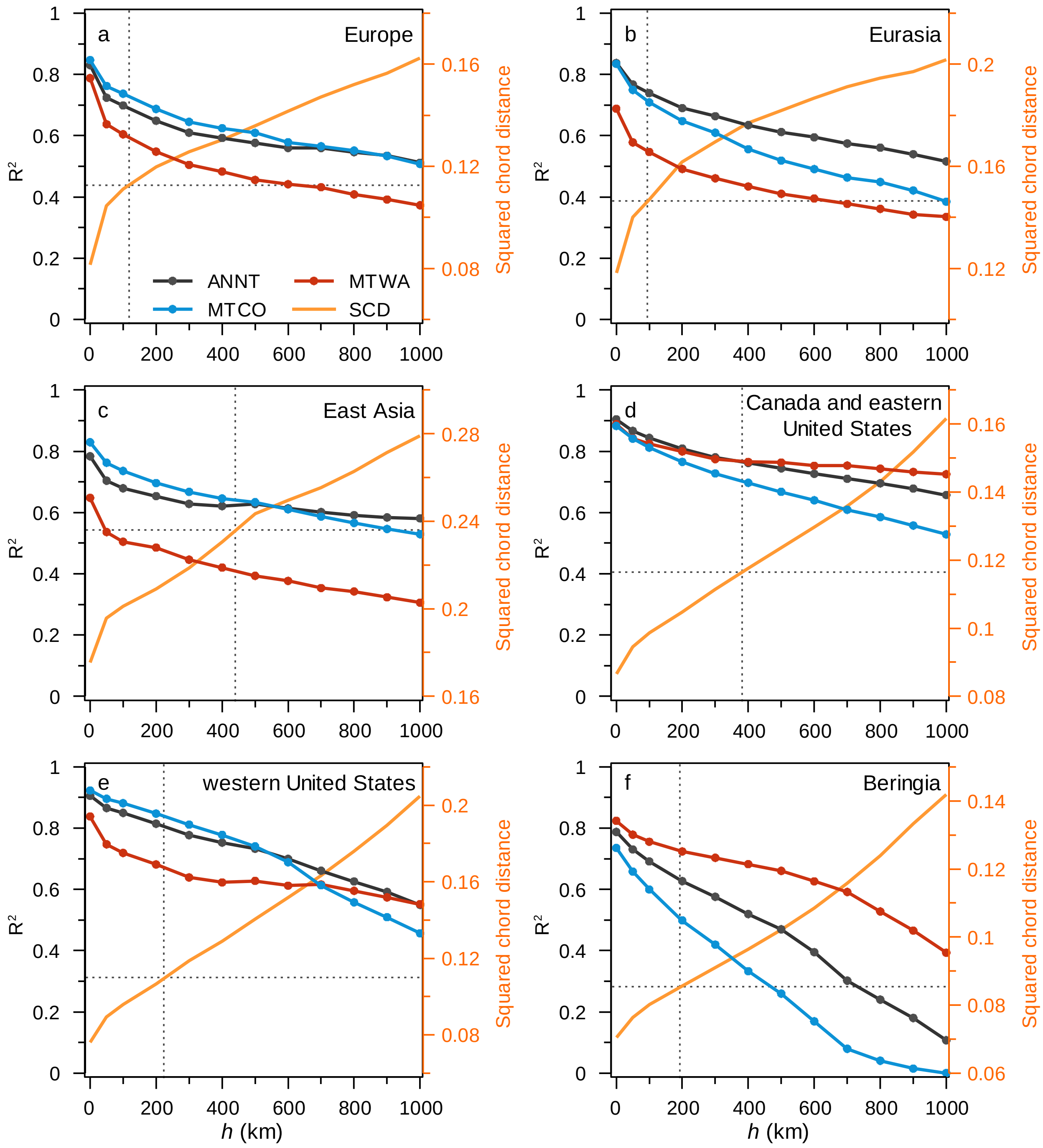

Supplementary Fig. 2. Model performance in *h*-block cross-validation experiments. Figures show the R^2 and median squared chord distance along with the changing distance *h* for Europe (**a**), Eurasia (**b**), East Asia (**c**), Canada and the eastern United States (**d**), the western United States (**e**) and Beriagia (**f**) regions. The horizontal dotted lines indicate the median squared chord distance for samples from the six regions during the Holocene. The vertical dotted lines indicate the selected *h* for the final evaluation of the model performance.

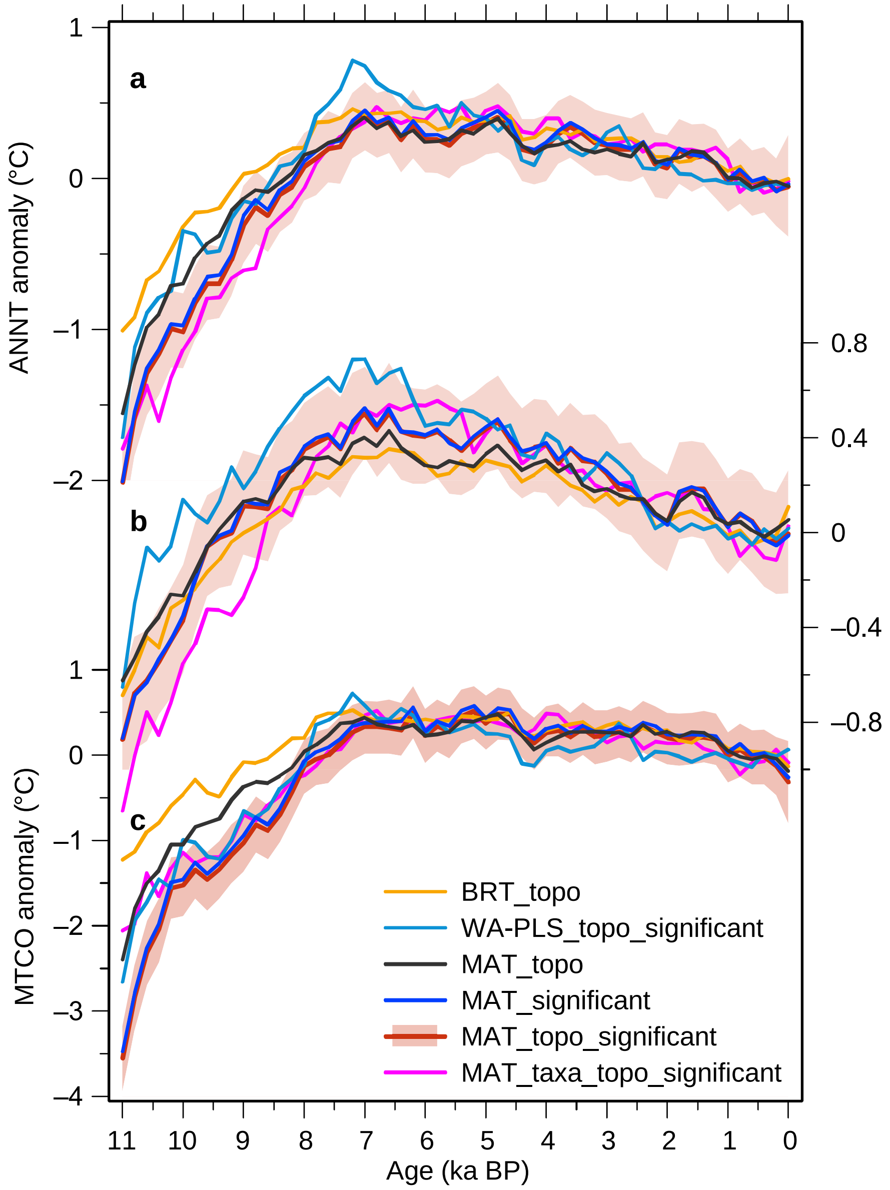

Supplementary Fig. 3. Influence of significance selection, isostatic correction and method choice on Holocene temperature reconstructions over the NH landmass. Figures show the results of annual (**a**), summer (**b**) and winter (**c**) temperatures using MAT based on PFT scores after significance selection and isostatic correction (red lines with 95% uncertainty bands), and the comparisons with the results without isostatic correction (blue lines), without significance selection (black lines), and the results using WA-PLS (teal lines), BRT (yellow lines), and MAT based on taxa data (magenta lines).

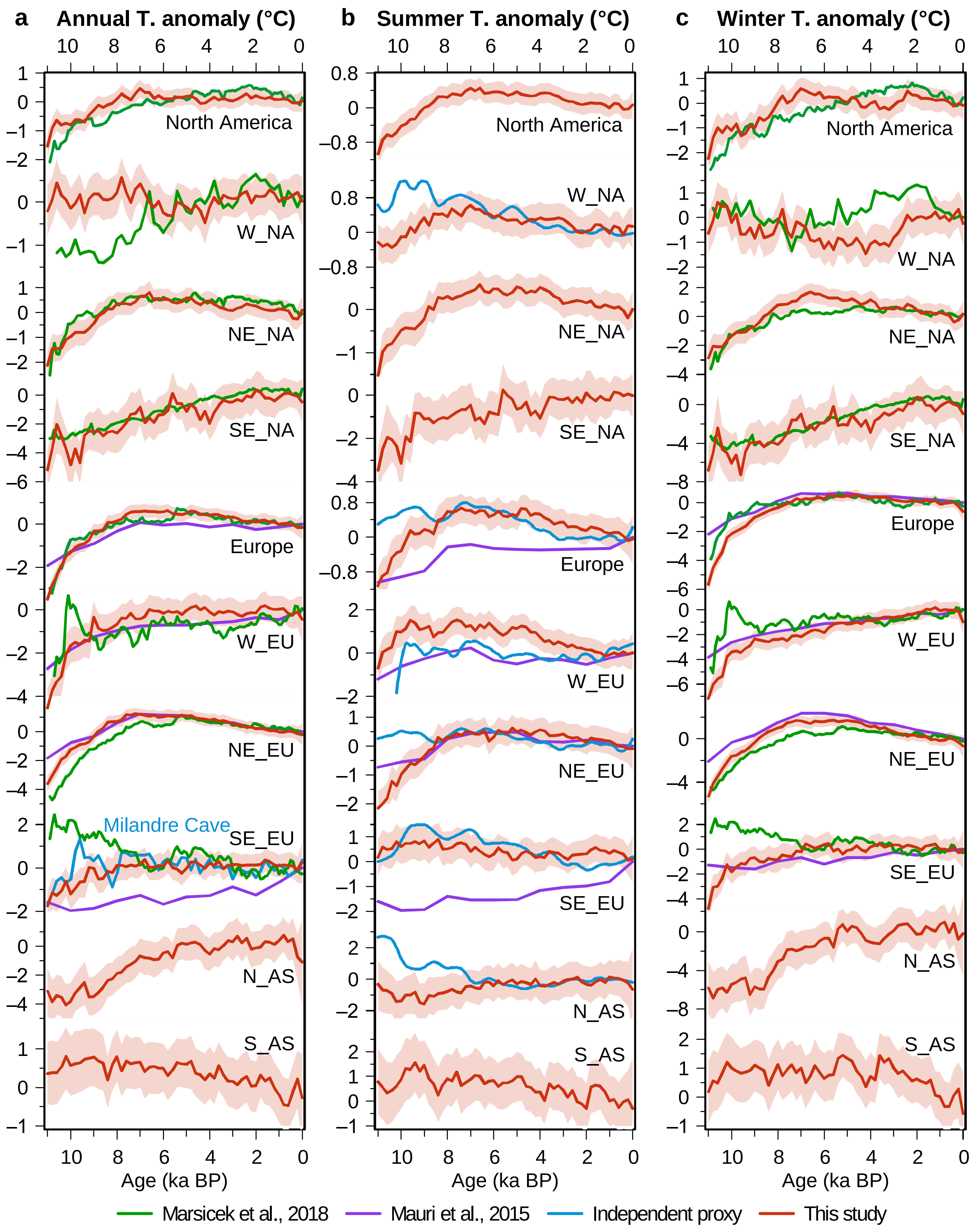
a
Annual T. anomaly (°C)
b
Summer T. anomaly (°C)
c
Winter T. anomaly (°C)
North America
W_NA
NE_NA
SE_NA
Europe
W_EU
NE_EU
SE_EU
Milandre Cave
N_AS
S_AS
Age (ka BP)
Marsicek et al., 2018
Mauri et al., 2015
Independent proxy
This study

Supplementary Fig. 4. Regional variability of temperature variations in the NH during the Holocene. Figures show variations of (**a**) annual, (**b**) summer and (**c**) winter temperatures during the Holocene. Regions: North America, including western North America (W_NA), northeastern North America (NE_NA) and southeastern North America (SE_NA); Europe, including western Europe (W_EU), northeastern Europe (NE_EU) and southeastern Europe (SE_EU); northern Asia (N_AS); and southern Asia (S_AS). The geographical ranges of the regions are the same as those in Fig. 2. Colours of lines indicate sources of data: red from this study, green from Marsicek et al.[1], purple from Mauri et al.[2], and teal from our chironomid-based compilations (see Methods) and Milandre Cave fluid inclusion temperature record[3].

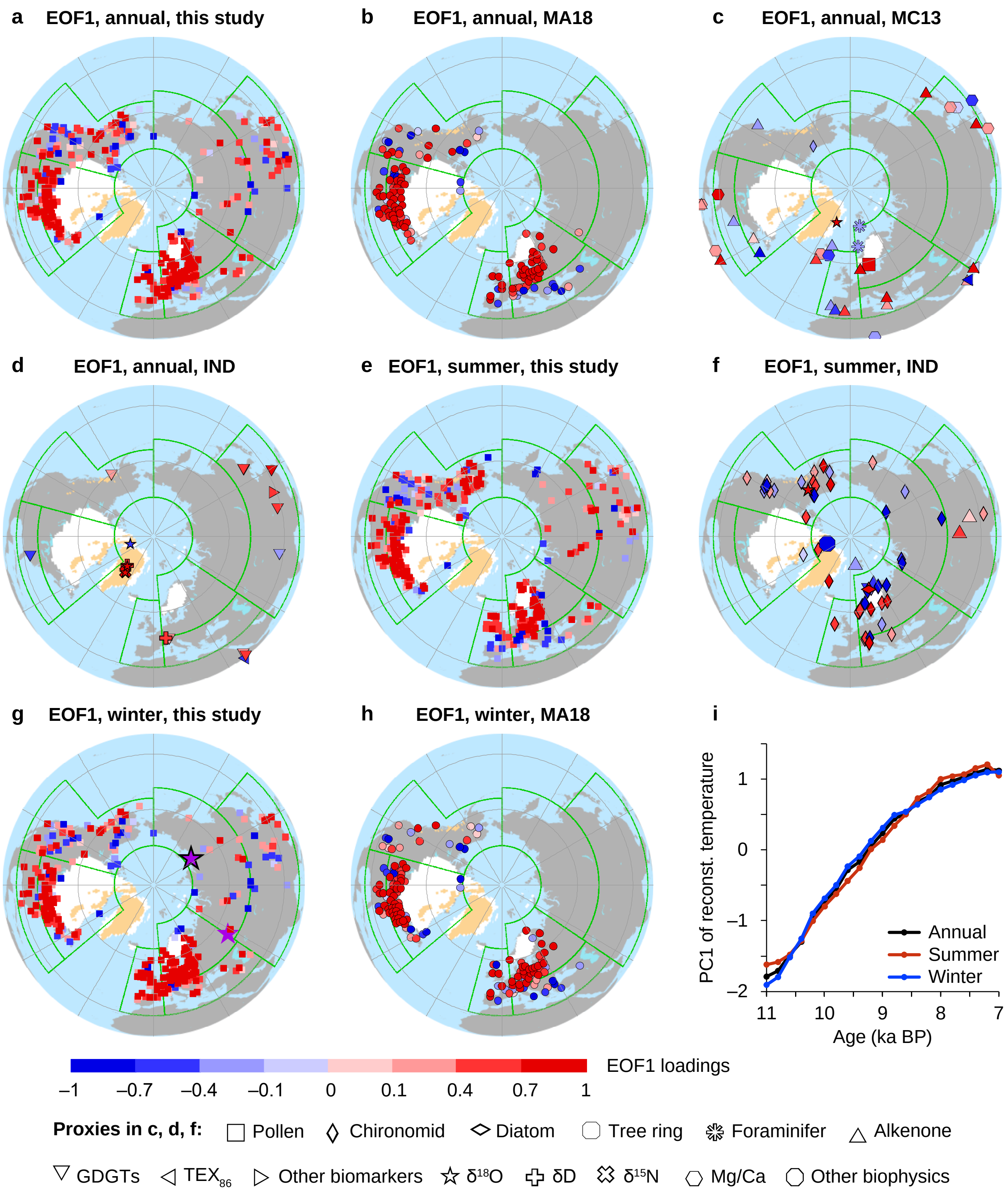

Supplementary Fig. 5. Comparison of spatial patterns of temperature changes during 11–7 ka BP. EOF1 patterns of annual temperature from (**a**) this study, (**b**) Marsicek et al.[1], (**c**) Marcott et al.[4], and (**d**) our independent compilations (Methods). EOF1 patterns of summer temperature from (**e**) this study and (**f**) independent compilations, and of winter temperature from (**g**) this

study and (**h**) Marsicek et al.[1]. **i**, PC1 of annual (black), summer (red) and winter (blue) temperature changes, which explain 57%, 50% and 58%, respectively, of the total variance. The symbols for the sites in (**c**), (**d**) and (**f**) correspond to the proxy categories shown in the bottom legend. The purple stars in (**g**) indicate the location of the KC[5] and Lena Delta[6] (with black border) sites. The green polygons enclose the same regions used in Fig. 2. The white and yellow shading indicates the ICE-7G ice sheet range at 11 and 7 ka BP, respectively[7].

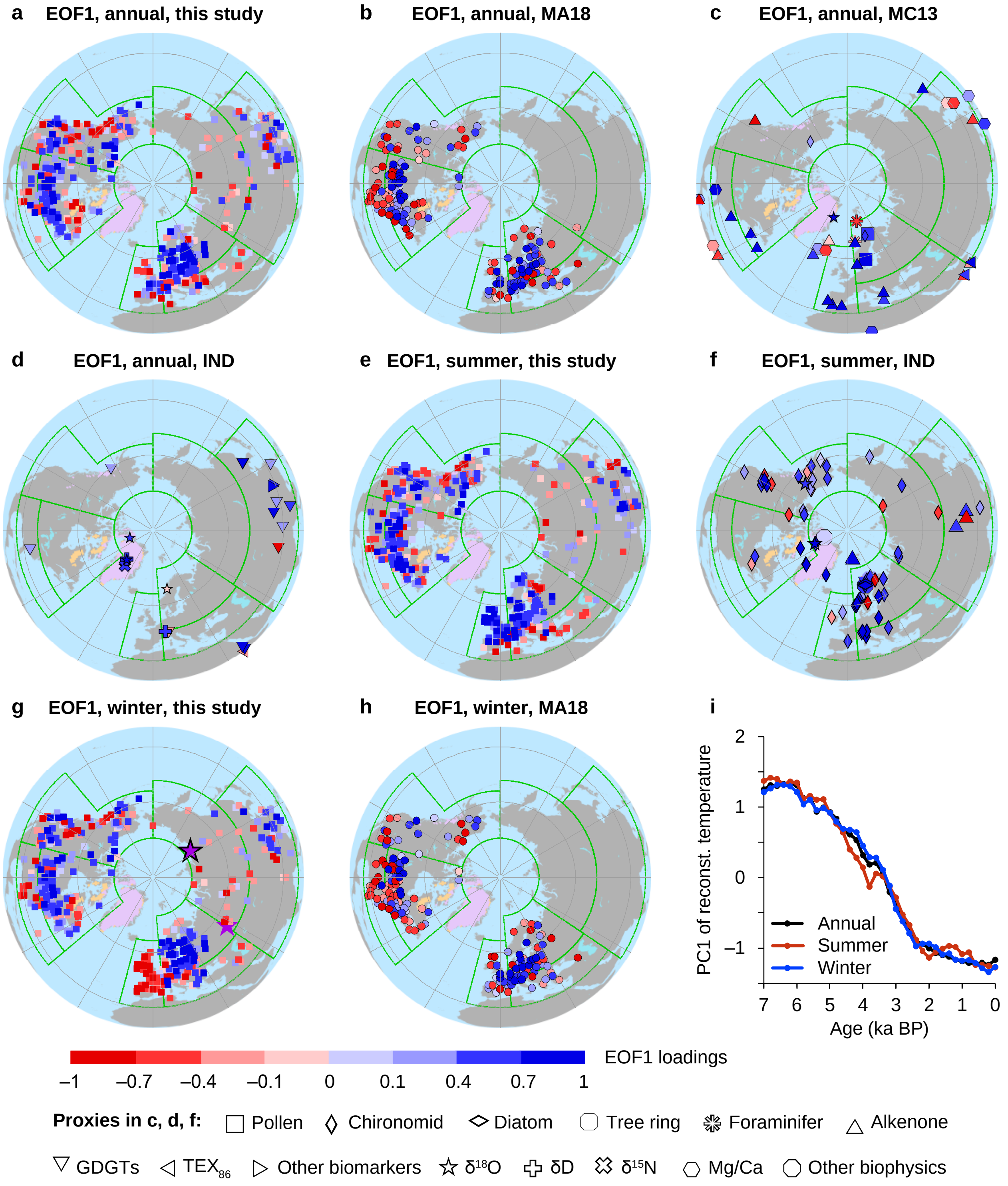

Supplementary Fig. 6. Comparison of spatial patterns of temperature changes during 7–0 ka BP. a–i, As Supplementary Fig. 5, but for 7–0 ka BP. EOF1 patterns of annual temperature from (**a**) this study, (**b**) Marsicek et al.[1], (**c**) Marcott et al.[4], and (**d**) our independent compilations (Methods). EOF1 patterns of summer temperature from (**e**) this study and (**f**) independent

compilations, and of winter temperature from (**g**) this study and (**h**) Marsicek et al.[1]. **i**, PC1 of annual (black), summer (red) and winter (blue) temperature changes. The percentages of the variance explained by PC1 are 35%, 33% and 41% for annual, summer and winter temperatures, respectively. The symbols for the sites in (**c**), (**d**) and (**f**) correspond to the proxy categories shown in the bottom legend. The purple stars in (**g**) indicate the location of the KC[5] and Lena Delta[6] (with black border) sites. The green polygons enclose the same regions used in Fig. 2. The yellow and pink shading indicates the ICE-7G ice sheet range at 7 ka BP and at the present, respectively[7].

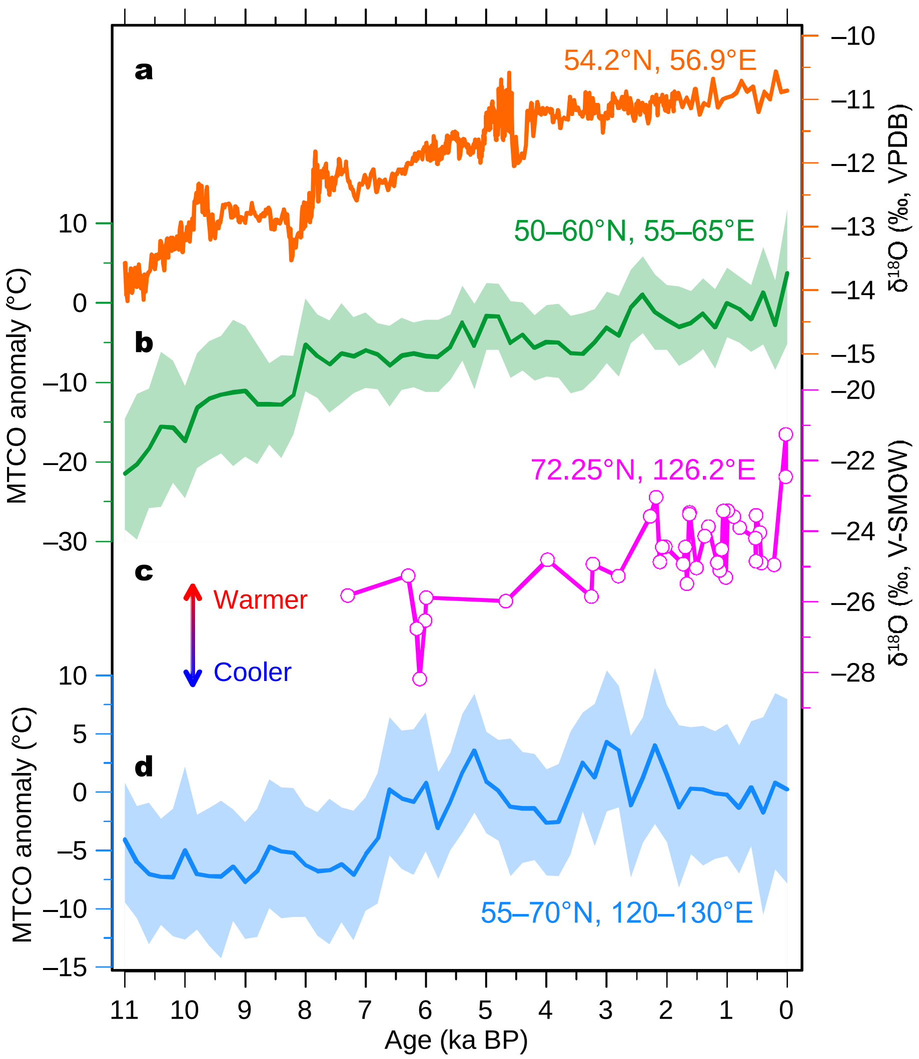

Supplementary Fig. 7. Holocene winter temperature records in Eurasia. The $\delta^{18}O$ values from the (**a**) KC stalagmite[5] and (**c**) Lena Delta ice wedge[6] records, and the corresponding pollen-based winter temperature stacks from sites near (**b**) KC and (**d**) Lena Delta. Continuous $\delta^{18}O$ enrichment at these two sites is thought to indicate long-term winter warming during the Holocene[5,6].

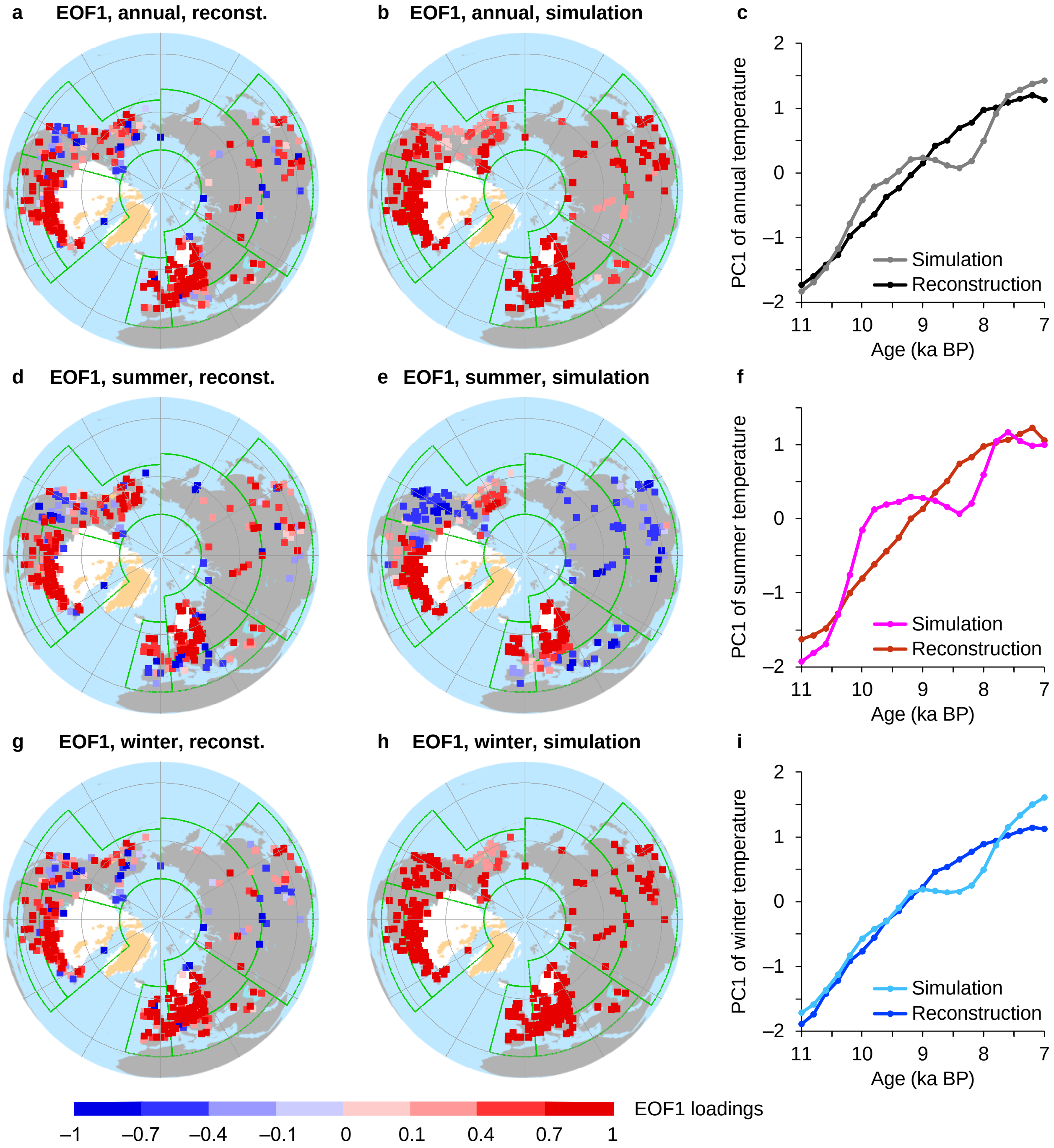

Supplementary Fig. 8. Model–data comparisons of annual and seasonal temperature patterns for 11–7 ka BP period. EOF1 patterns of pollen-based and CCSM3-simulated annual (**a** and **b**), summer (**d** and **e**), and winter (**g** and **h**) temperature changes, and PC1 series for reconstructions and simulations of annual (**c**), summer (**f**), and winter temperatures (**i**). PC1 of annual, summer and winter temperature changes explain 58%, 52% and 58%, respectively, of the total variance of the reconstructions, and 92%, 76% and 92%, respectively, of the simulations.

The white and yellow shading indicates the ICE-7G ice sheet range at 11 and 7 ka BP, respectively[7].

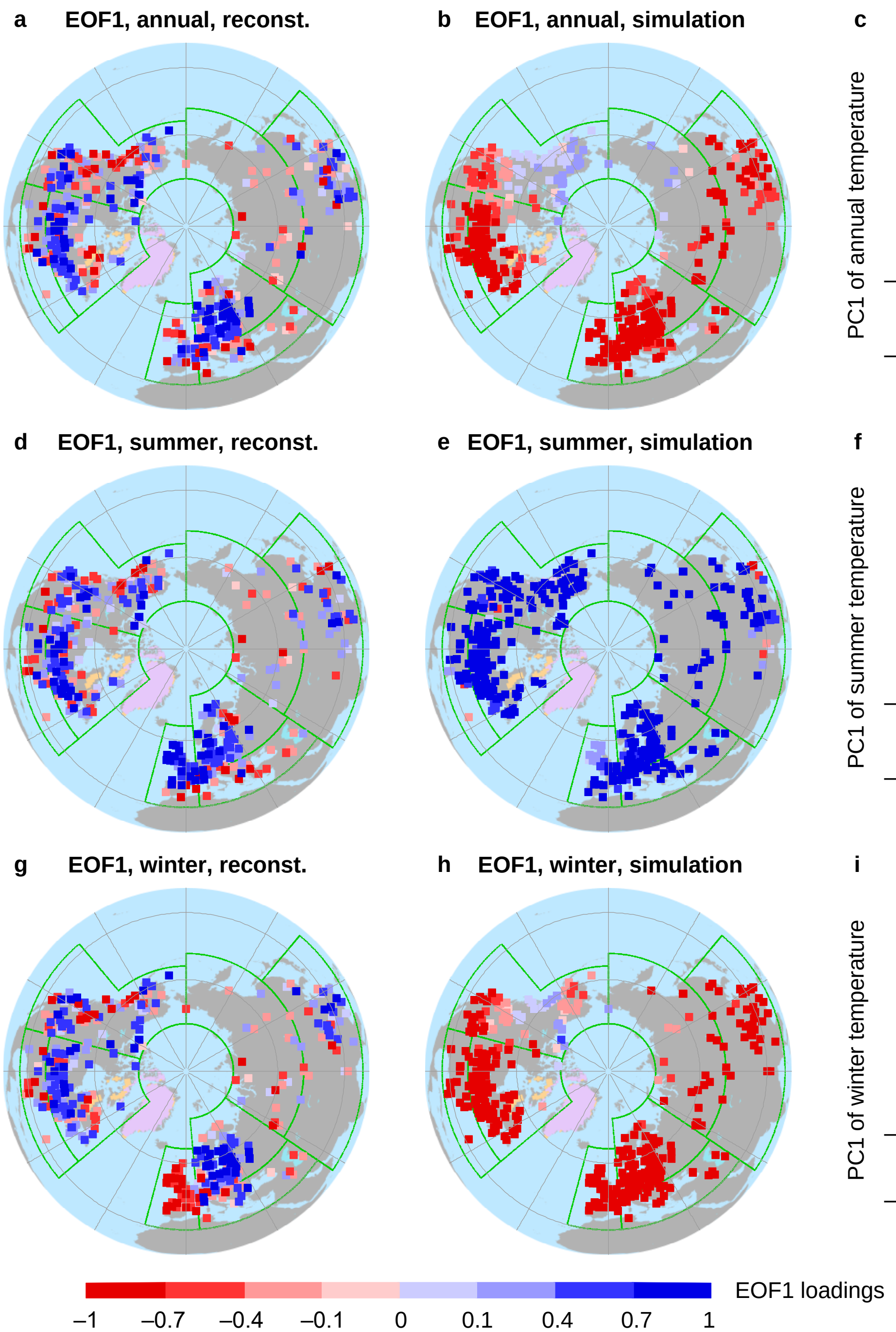

Supplementary Fig. 9. Model–data comparisons of annual and seasonal temperature patterns for 7–0 ka BP period. a–i, As Supplementary Fig. 8 but for 7–0 ka BP. EOF1 patterns of pollen-based and CCSM3-simulated annual (**a** and **b**), summer (**d** and **e**), and winter (**g** and **h**) temperature changes, and PC1 series for reconstructions and simulations of annual (**c**), summer (**f**), and winter temperatures (**i**). PC1 of annual, summer and winter temperature changes explain 36%, 31% and 42%, respectively, of the total variance of the reconstructions, and 47%, 86% and

66%, respectively, of the simulations. The yellow and pink shading indicates the ICE-7G ice sheet range at 7 and 0 ka BP, respectively[7].

Supplementary Table 1. Contributions of MTCO and MTWA to the variation of modern PFT score data based on RDA. *R* is Pearson correlation coefficient for MTCO and MTWA in the calibration datasets. *λ1/λ2*, the ratio of the eigenvalues of the constrained and the first unconstrained axes in single-variable RDA runs, represents the relative explanatory power of the selected variable. *Explained* and *Unique* are the whole and unique proportions of total variation explained by each variable in the partial RDA. Correspondingly, the whole and shared proportions for both variables are displayed in the following row (*Common*). The significance (*p* value) of each variable with respect to variation explaining was assessed using Monte Carlo permutation tests (1000 permutations). The RDA was run separately in the six regions used for the PFT calculations.

Region	Variable	R	λ1/λ2	Explained (%)	Unique (%)	Covariance prop. (%)	p
Europe	MTCO		0.92	14.8	7.9	46.5	0.001
	MTWA		0.60	11.3	4.4	60.8	0.001
	Common	0.67		19.2	6.9	35.8	
Eurasia	MTCO		0.51	9.9	5.8	40.9	0.001
	MTWA		0.31	6.3	2.3	64.1	0.001
	Common	0.57		12.1	4.0	33.3	
East Asia	MTCO		0.68	13.4	10.1	24.8	0.001
	MTWA		0.37	7.9	4.6	42.0	0.001
	Common	0.54		18.0	3.3	18.5	
Canada and the eastern United States	MTCO		1.15	20.4	5.4	73.7	0.001
	MTWA		1.02	20.5	5.5	73.1	0.001
	Common	0.76		25.9	15	58.0	
western United States	MTCO		0.82	17.9	10.2	43.2	0.001
	MTWA		0.87	16.5	8.8	46.9	0.001
	Common	0.64		26.7	7.7	29.0	
Beringia	MTCO		0.57	13.5	13.3	1.5	0.001
	MTWA		0.36	10.4	10.2	2.0	0.001
	Common	0.35		23.7	0.2	0.9	

Supplementary Table 2. MAT performance for temperature reconstructions. For each climate variable, the coefficient of determination (R^2) and the root-mean-square error of prediction (RMSEP, °C) were calculated by leave-one-out (LOO) and *h*-block (HB) cross validation. The number of modern analogues was determined from the lowest RMSEP among the 5–7 closest analogues. The distance (*h*, km) for HB was determined by the squared chord distance through a series of experiments (see Methods and Supplementary Fig. 2).

**Region**	**Variable**	**R^2, LOO**	**RMSEP, LOO**	**R^2, HB**	**RMSEP, HB**	**No. analogue**	**Thre-shold**	***h***
Europe	ANNT	0.85	2.0	0.70	2.8	6	0.2	100
	MTCO	0.87	2.4	0.74	3.3	6		
	MTWA	0.81	2.0	0.61	2.9	7		
Eurasia	ANNT	0.85	3.1	0.74	4.0	7	0.3	100
	MTCO	0.85	4.5	0.72	6.3	7		
	MTWA	0.69	2.8	0.54	3.5	6		
East Asia	ANNT	0.80	3.4	0.62	4.8	7	0.5	400
	MTCO	0.86	4.2	0.65	6.5	5		
	MTWA	0.68	3.7	0.42	5.0	7		
Canada and the eastern United States	ANNT	0.91	2.5	0.76	4.0	7	0.3	400
	MTCO	0.89	3.8	0.70	6.2	7		
	MTWA	0.89	1.9	0.76	2.8	7		
western United States	ANNT	0.91	2.2	0.81	3.3	7	0.3	200
	MTCO	0.93	2.8	0.85	4.3	7		
	MTWA	0.85	2.1	0.68	3.1	7		
Beringia	ANNT	0.80	2.4	0.63	3.3	7	0.3	200
	MTCO	0.75	3.7	0.50	5.3	7		
	MTWA	0.84	1.5	0.72	2.0	7		

Supplementary References:

1. Marsicek, J., Shuman, B. N., Bartlein, P. J., Shafer, S. L. & Brewer, S. Reconciling divergent trends and millennial variations in Holocene temperatures. *Nature* **554**, 92–96 (2018).
2. Mauri, A., Davis, B. A. S., Collins, P. M. & Kaplan, J. O. The climate of Europe during the Holocene: a gridded pollen-based reconstruction and its multi-proxy evaluation. *Quat. Sci. Rev.* **112**, 109–127 (2015).
3. Affolter, S. et al. Central Europe temperature constrained by speleothem fluid inclusion water isotopes over the past 14,000 years. *Sci. Adv.* **5**, v3809 (2019).
4. Marcott, S. A., Shakun, J. D., Clark, P. U. & Mix, A. C. A reconstruction of regional and global temperature for the past 11,300 years. *Science* **339**, 1198–1201 (2013).
5. Baker, J. L., Lachniet, M. S., Chervyatsova, O., Asmerom, Y. & Polyak, V. J. Holocene warming in western continental Eurasia driven by glacial retreat and greenhouse forcing. *Nat. Geosci.* **10**, 430–435 (2017).
6. Meyer, H. et al. Long-term winter warming trend in the Siberian Arctic during the mid- to late Holocene. *Nat. Geosci.* **8**, 122–125 (2015).
7. Roy, K. & Peltier, W. R. Relative sea level in the Western Mediterranean basin: A regional test of the ICE-7G_NA (VM7) model and a constraint on late Holocene Antarctic deglaciation. *Quat. Sci. Rev.* **183**, 76–87 (2018).
