# Supplementary material for: Holocene seasonal temperature evolution and spatial variability over the Northern Hemisphere landmass
Source: Nat Commun. 2022 Sep 10;13:5334. doi: 10.1038/s41467-022-33107-0 (PMC9464234; doi:10.1038/s41467-022-33107-0)
Supplement: Supplementary file 3 — Description of Additional Supplementary Files [file 41467_2022_33107_MOESM3_ESM.pdf]

Description of Additional Supplementary Files

File Name: Supplementary Data 1
Description: Site information for pollen and independent proxy records.

File Name: Supplementary Data 2
Description: Plant Functional Type (PFT) – taxa matrix.

File Name: Supplementary Data 3
Description: Model performance for temperature reconstructions.
